# Thioacetamide Additive Homogenizing Zn Deposition Revealed by In Situ Digital Holography for Advanced Zn Ion Batteries

**DOI:** 10.1007/s40820-023-01310-3

**Published:** 2024-02-15

**Authors:** Kaixin Ren, Min Li, Qinghong Wang, Baohua Liu, Chuang Sun, Boyu Yuan, Chao Lai, Lifang Jiao, Chao Wang

**Affiliations:** 1 School of Chemistry and Materials Science, Jiangsu Normal University, Xuzhou, 221116 Jiangsu People’s Republic of China; 2 Jiangsu Key Laboratory of Advanced Laser Materials and Devices, School of, Physics and Electronic Engineering, Jiangsu Normal University, Xuzhou, 221116 Jiangsu People’s Republic of China; 3 Key Laboratory of Advanced Energy Materials Chemistry (Ministry of Education), Nankai University, 300071 Tianjin, People’s Republic of China

**Keywords:** Digital holographic microscopy, In situ observation, Electrode/electrolyte interface, Zn dendrites, Screening electrolyte additives

## Abstract

Zinc ion batteries are considered as potential energy storage devices due to their advantages of low-cost, high-safety, and high theoretical capacity. However, dendrite growth and chemical corrosion occurring on Zn anode limit their commercialization. These problems can be tackled through the optimization of the electrolyte. However, the screening of electrolyte additives using normal electrochemical methods is time-consuming and labor-intensive. Herein, a fast and simple method based on the digital holography is developed. It can realize the in situ monitoring of electrode/electrolyte interface and provide direct information concerning ion concentration evolution of the diffusion layer. It is effective and time-saving in estimating the homogeneity of the deposition layer and predicting the tendency of dendrite growth, thus able to value the applicability of electrolyte additives. The feasibility of this method is further validated by the forecast and evaluation of thioacetamide additive. Based on systematic characterization, it is proved that the introduction of thioacetamide can not only regulate the interficial ion flux to induce dendrite-free Zn deposition, but also construct adsorption molecule layers to inhibit side reactions of Zn anode. Being easy to operate, capable of in situ observation, and able to endure harsh conditions, digital holography method will be a promising approach for the interfacial investigation of other battery systems.

**Supplementary Information:**

The online version contains supplementary material available at 10.1007/s40820-023-01310-3.

## Highlights

- Digital holography can realize the in situ observation of electrode/electrolyte interface and provide dynamic evolution information of the liquid phase of electrode, which is both efficient and effective in investing the interficial electrochemical mechanism and screening electrolyte additives.
- Thioacetamide electrolyte additive effectively enhances the electrochemical performance of Zn anode by regulating the interficial ion flux to induce dendrite-free Zn deposition and constructing adsorption molecule layers to inhibit side reactions.

**Supplementary Information:**

The online version contains supplementary material available at 10.1007/s40820-023-01310-3.

## Introduction

Aqueous zinc-ion batteries (ZIBs) have attracted overwhelming attention due to their advantages of non-flammability, low cost, high theoretical capacity (820 mAh g^−1^) and low electrochemical potential (−0.76 V, vs. SHE). Unfortunately, their application as energy storage systems is seriously bottlenecked by the poor electrochemical performance of the Zn anode [1, 2]. Firstly, the undesirable dendrite growth occurring on the anode during the plating process may pierce the separator and result in short circuit [3]. Secondly, unavoidable side reactions (mainly including self-corrosion and hydrogen evolution) occurring during the long-term cycling process may cause the passivation of the anode and result in its inefficient utilization [4].

To overcome these shortcomings, several strategies, such as structural construction, surface modification and electrolyte optimization, have been proposed to enhance the cycling stability and coulombic efficiency (CE) of Zn anodes [5, 6]. Among these methods, the last category has been proved to be simple and effective in depressing the dendrite growth [7]. So far, various additives, such as organic molecules [8, 9], high-concentration metal salts [10], organic electrolytes [11] have been employed for the electrolyte optimization. However, the traditional electrochemical measurements used in the search for feasible additives are laborious and time-consuming for they mainly depend on the trial-and-error method. Rapid screening for effective electrolyte additives from huge database with high accuracy and good reliability is a great challenge at this stage.

It is known that Zn dendrites are mainly formed during the dynamic Zn plating process, which involves early-stage nucleation and subsequent growth process. The plating quality largely depends on the former stage because Zn nuclei strongly interfere with the electric field and ion concentration distribution at the electrode/electrolyte interface, which determines the growth of the Zn layer [12]. Therefore, the detection of the dynamic changes of the concentration diffusion layer and the concentration gradient at the electrode/electrolyte interface during the plating process, especially at the early-stage, is particularly important for evaluating the Zn protection effect of electrolyte additives. At present, various advanced in situ and ex situ techniques have been employed to reveal the evolution of the Zn anode during the plating process [13]. X-ray diffraction (XRD) [14, 15], Fourier transform infrared spectroscopy (FTIR) [16, 17], Raman spectroscopy (Raman) [18, 19], X-ray photoelectron spectroscopy (XPS) [20, 21] and X-ray absorption fine structure (XAFS) [22, 23] can provide valuable information concerning the crystal and chemical structure changes of the electrode during the plating/striping process. Visualization techniques, such as scanning electron microscopy (SEM) [24–26], in situ transmission electron microscopy (TEM) [27, 28], in situ optical microscopy [29], X-ray microscopy [30] and in situ atomic force microscopy (AFM) [31, 32] can record the morphology evolution of Zn anodes in the nucleation and growth process and provide direct evidence of Zn dendrites. All these methods have contributed a lot in investigating the Zn dendrite growth, evaluating the suppression performance and comprehending the suppression mechanism. Unfortunately, under most circumstances, they set high and strict demands on test conditions, which seriously obstacle their wide application. Moreover, they focus solely on the solid electrode, ignoring the real-time monitoring of the changes of the electrolyte side at the electrochemical interface, which is also important in Zn deposition.

**Scheme 1:**
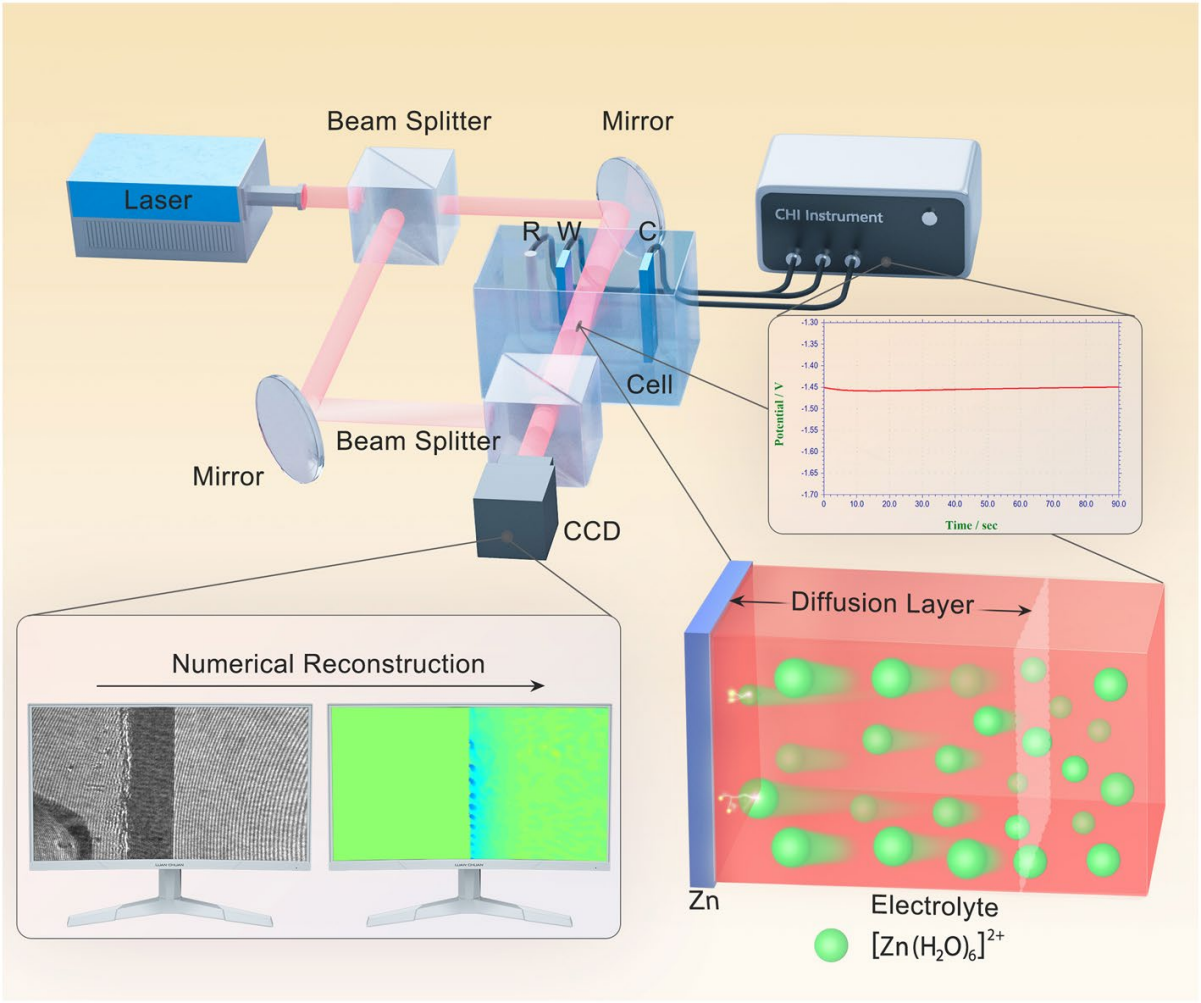
The observation setup of the DHM system and a schematic diagram of the dynamic Zn deposition at the electrode/electrolyte interface. *R* reference electrode, *C* counter electrode, *W* working electrode, *CCD* charge-coupled device

The digital holographic microscopy (DHM) is a powerful optical technique for the study of microscopic samples via sensing the variations of their refractive index. The experimental setup of the holographic recording system is shown in Fig. S1 and schematically illustrated in Scheme 1. The optical part is a Mach–Zehnder interferometer with a wavelength of 632.8 nm, which is generated by a He–Ne laser. Interferograms corresponding to various states of the electrode/electrolyte interface are recorded in situ with a CCD camera and then transformed into phase maps through numerical reconstruction. During the electrochemical measurements, DHM can record the changes of amplitude and phase at the electrode/electrolyte interface in transparent solutions and reveal the dynamic evolution of the concentration gradient and the diffusion layer [33, 34]. It also possesses the advantages of high temporal resolutions, noncontact and nondestructive working principle, and low setup requirements [35, 36]. It has been applied in the field of metal corrosion and protection, capturing the interfacial changes of the pitting corrosion with high precision [37, 38]. In our previous reports, DHM was employed to verify the formation of solid electrolyte interphase film on graphite anode in Li-ion batteries and to confirm the migration of polysulfide in Li–S batteries [39, 40]. Therefore, DHM is a reliable method for in situ observation of electrochemical processes, especially non-uniform reactions taking place at the electrode/electrolyte interface.

Herein, DHM is introduced to investigate the initial Zn dendrite growth process in various electrolytes. Combined with electrochemical methods and morphology characterization, the effect of current density and electrolyte additives on dendrite growth was further investigated with the digital holographic interface imaging. Moreover, the effect of thioacetamide (TAA) additive on the electrochemical performance of Zn anode was pre-estimated by DHM and further verified by electrochemical measurements. The application of DHM will not only increase the efficiency and accuracy in addictive screening, but also help to clarify the growth mechanism of Zn dendrites.

## Experimental Procedures

### DHM Measurements

During the galvanostatic Zn deposition, digital holographic measurements were synchronously carried out in a three-electrode system, which was assembled in a transparent optical cell. The temporal-resolution of the DHM system is defined by the CCD camera (Prosilica GT 1910). The max frame rate at full resolution is 57.5 fps. Herein, it is set to be 50 fps and the real temporal-resolution of the DHM measurement is 20 ms. The cell was a cube with a side length of 5 cm. The volume of electrolyte was about 60 mL. Both the working and the counter electrodes were Zn foil, of which the thickness is 0.3 mm and the purity is 99.99%. The distance between them was about 2 cm. The working electrode was sealed with epoxy resin and punched into sheets with an area of 0.2 × 1 cm^2^. Hg_2_SO_4_/Hg electrode served as the reference electrode. Before each electrochemical experiment, the working electrode was abraded with a series of wet sandpaper with different grit sizes (1200#, 2500#, 4000# and 7000#). Subsequently, it was cleaned with distilled water and ethanol in an ultrasonic bath. In a three-electrode system, the electrode, observed by DHM, was plated for 1.5 min at different current densities (0.1, 0.5, 1.0, 2.0, 4.0 mA cm^−2^) using an electrochemical workstation (CHI660E, CH Instruments) at room temperature.

### Calculations

Density functional theory (DFT) calculations in this work were carried out using the CP2K package (version-2022.1) [41] and ORCA (version: 5.0.3) [42], and the details for DFT calculations were displayed in Supporting Information. The LUMO (Lower Unoccupied Molecular Orbital) and HOMO (Highest Occupied Molecular Orbital) orbitals were analyzed by MULTIWFN [43, 44]; and VMD software [45] was used to draw these orbitals.

The solvation structures of 2 M ZnSO_4_ solution and 2 M ZnSO_4_-10 mM TAA solution were simulated by molecular dynamics, with the GROMACS software package (2020.6 version) [46, 47]. The details were displayed in Supporting Information. The simulation results were visualized and analyzed with VMD.

### Characterization

The morphology of the samples was characterized by a field emission scanning electron microscope (SEM, SU8010) and AFM (Bruker Dimension Icon). The elemental composition of the electrode was analyzed by XRD (Bruker D8 ADVANCE), energy dispersive X-ray spectroscopy (EDS, linked with SEM), and XPS (Thermo Scientific ESCALAB 250Xi) measurements. The H magnetic resonance imaging (NMR) spectroscopy is performed on a Bruker Avance NEO (400 MHz). The morphology evolution of Zn anode during the plating process was detected by the optical microscope (NIKON SMZ1270). Ionic conductivity of the electrolytes were tested using conductivity meter produced by Shanghai Shiyi Precision Instruments Co, ltd.).

### Fabrication of Symmetric Cells and Zn–V_2_O_5_ Full Cells

Blank electrolyte, 2 M ZnSO_4_ solution, was prepared by dissolving ZnSO_4_ powder (Sinopharm Company) into the deionized water. Dimethyl sulfoxide (DMSO)-based electrolyte was obtained in the same way by using H_2_O/DMSO (volume ratio = 1:1) instead of water. TAA-based electrolytes were prepared by dissolving different amounts of TAA into the blank electrolyte. The concentrations of TAA were 5, 10, and 15 mM, and the electrolytes were marked as ZnSO_4_-5 mM TAA, ZnSO_4_-10 mM TAA and ZnSO_4_-15 mM TAA, respectively.

Two pieces of Zn foils with a thickness of 100 μm were used as the electrodes for symmetric cells. Two different electrolytes (2 M ZnSO_4_ and 2 M ZnSO_4_ with TAA) were added into the coin cell with a piece of glass fiber (Whatman, GF/D-90 mm) as a separator.

Full cells were assembled via Zn anode and V_2_O_5_ cathode. The cathode was prepared by mixing V_2_O_5_ powder, conductive carbon, and polytetrafluoroethylene (PTFE) in a weight ratio of 7:2:1 with ethanol as a solvent. Then the slurry was pressed into thin sheets and cut into pieces. The average mass loading of active material was around 1.0 mg cm^−2^.

### Electrochemical Measurements

The performances of Zn//Zn symmetric cells and Zn//V_2_O_5_ full cells were collected by using CR2032 coin-type cells on a battery test system (LAND CT2001A). For symmetric cells, constant current densities were applied ranging from 1 to 4 mA cm^−2^ and the charging and discharging times were both set to be 1 h. For Zn//V_2_O_5_ cells, the rate performance and cycling performance were investigated from 0.2 to 1.6 V under various current densities (range from 0.2 to 2 A g^−1^) to validate the practicality and feasibility of TAA electrolyte additive in application. Coulombic efficiency (CE) measurements were carried out on asymmetrical Zn//Cu cells. In addition, all cyclic voltammetry (CV), Tafel curves and electrochemical impedance spectroscopy (EIS) data were obtained on an electrochemical workstation (CHI 760E). CV tests were conducted in the voltage range of 0–2.0 V for full cells. Linear polarization curves were recorded by voltage scanning between −0.3 and 0.3 V vs the open circuit potential at 1 mV s^−1^. EIS experiment was carried out with a frequency range from 10^5^ to 10^–2^ Hz with the perturbation of 5 mV. Chronoamperometry (CA) measurement was conducted with the constant potential of 25 mV for 1000 s in symmetric cells.

## Results and Discussion

### Feasibility of DHM for the In Situ Observation of Zn Anode

**Fig. 1:**
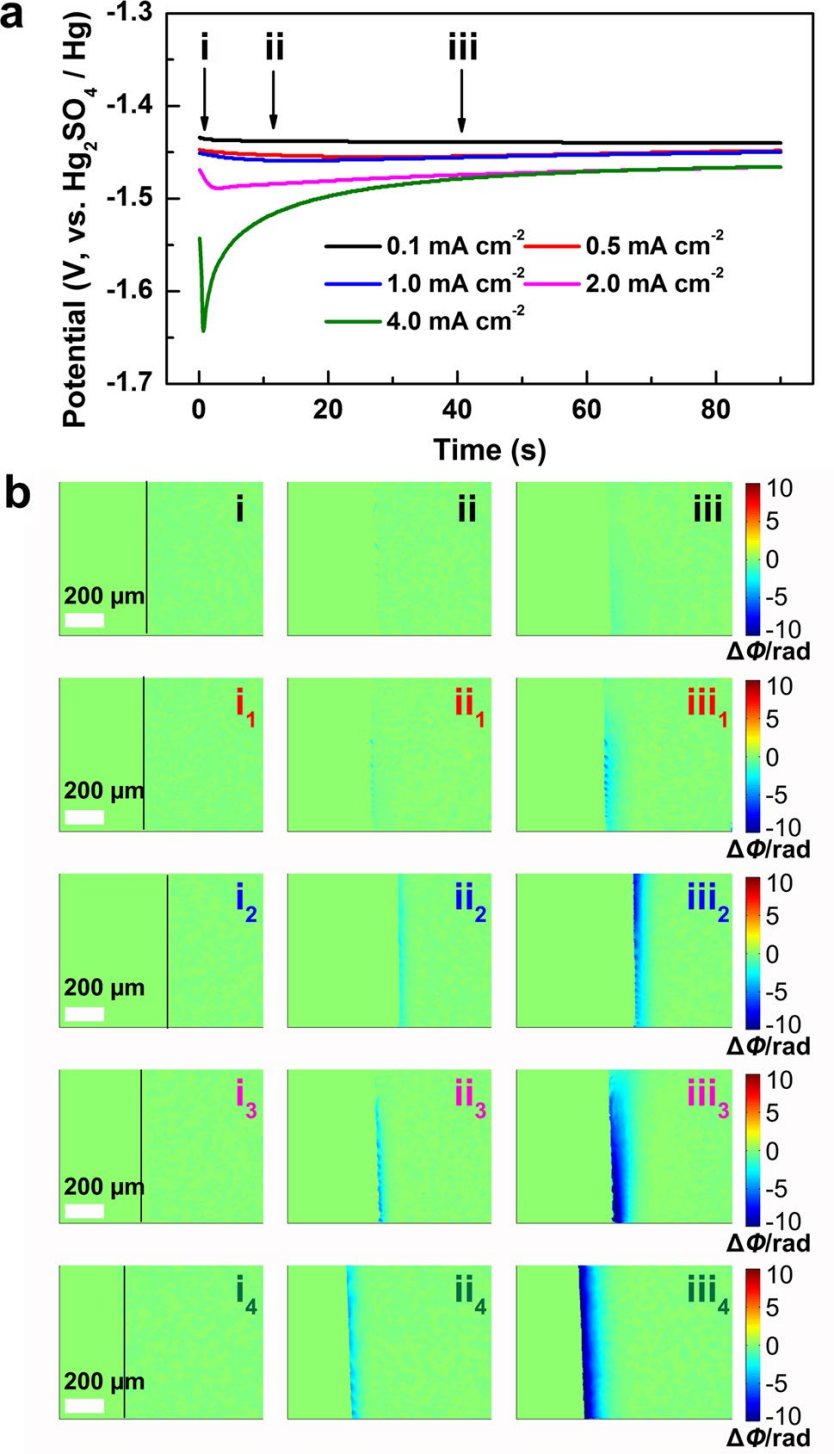
**a** The voltage profiles of galvanostatic Zn deposition at different current densities in 2 M ZnSO_4_ electrolyte. **b** The phase maps corresponding to points (i–iii) in (**a**). (i–iii): 0.1 mA cm^−2^, (i_1_–iii_1_): 0.5 mA cm^−2^, (i_2_–iii_2_): 1.0 mA cm^−2^, (i_3_–iii_3_): 2.0 mA cm^−2^, (i_4_–iii_4_): 4.0 mA cm^−2^

To investigate the feasibility of DHM, the dynamic evolution of the electrode/electrolyte interface during the initial Zn deposition under various current densities were detected by DHM in a three-electrode system. Figure 1a shows the initial voltage profiles at various current densities from 0.1 to 4.0 mA cm^−2^ in 2 M ZnSO_4_ aqueous solution. It can be seen that the nucleation overpotential shifts negatively with the increase of the current density (−1.44 V for 0.1 mA cm^−2^, −1.64 V for 4.0 mA cm^−2^). It is reported that higher current density corresponds to higher nuclei density with a smaller size of Zn deposits, and lower areal capacity renders smaller Zn flakes, which contributes to the long cycle life of the Zn anode [48].

As shown in Movie S1, when Zn is electrodeposited, the change of the ion concentration on the liquid side of the electrode/electrolyte interface results in the changes in the refractive index of the electrolyte ($\Delta n)$, and interference fringes will appear on the hologram when the following condition is satisfied:

1 $${\Delta }n = (2i + 1)\lambda /2d$$

where* i* is an integer representing the order of the fringe, *λ* is the wavelength of the laser light, and d is the thickness of the solution layer in which the concentration changes in the direction of the optical axis. After the numerical reconstruction, the interference fringes are conversed to the phase maps, which can visually and dynamically present the relevant ion concentration evolution of the diffusion layer during the deposition process. At this very moment, the relationships among the concentration change of the electrolyte ($\Delta C$), the refractive index variation ($\Delta n$) and the phase difference ($\Delta \Phi$) at the electrode/electrolyte interface are as follows:

2 $$\Delta C = k\Delta n = (k\lambda /2\pi d)\Delta \Phi$$

where $k$ is a constant indicating the linear change between the solution concentration and the refractive index. In the phase maps, the green area means that $\Delta \Phi$ = 0, $\Delta$ *C* = 0, indicating the concentration remains unchanged. The blue area means that $\Delta \Phi$ < 0, $\Delta$ *C* < 0, indicating the concentration decrease, whereas the yellow or the red means that $\Delta \Phi$ > 0, $\Delta$ *C* > 0, indicating the concentration increase.

Figure 1b shows the corresponding phase distributions at the selected deposition times of 0, 10, and 40 s at various current densities, as marked in Fig. 1a. The black lines in Fig. 1bi, i_1_, i_2_, i_3_, i_4_) represent the electrode/electrolyte interface; the left side represents the electrode and the right the electrolyte. As shown in Fig. 1bii, at the current density of 0.1 mA cm^−2^, weak concentration decrease is observed at the deposition time of 10 s, indicating the conversion of Zn^2+^ to Zn during the initial deposition process. The blue area expands along with the deposition time; however, a uniform diffusion layer is not wholly formed till 40 s at such a low current density. Figure 1bii_1_ and iii_1_ display the phase distributions at the current density of 0.5 mA cm^−2^, which presents obviously the decrease of interfacial concentration, corresponding to the accelerated Zn deposition at a higher current density. It is worth noting that faint blue area starts to emerge at the interface at 10 s. With the deposition going on and the deepening of the blue, dots of dark blue become more and more obvious, indicating inhomogenous ion consumption. This phenomenon is known as the “tip effect”, which means that uneven deposition occurs on the Zn anode during the initial nucleation process, then leads to the electron accumulation and finally results in dendrite growth on the anode. Figure 1bii_2_–iii_2_, ii_3_–iii_3_ and ii_4_–iii_4_ display the interfacial concentration changes at higher current densities of 1.0, 2.0, and 4.0 mA cm^−2^. More obvious and homogenous concentration decrease is detected with the increase of the current density, indicating fast and flat Zn deposition occurs. Figure 1biii_4_ illustrates that an even diffusion layer is formed after 40 s deposition at the current density of 4.0 mA cm^−2^, which is beneficial for the formation of uniform Zn deposition. Therefore, the digital holographic measurements confirm that with the increase of the current density, the uniform diffusion layer can be formed in short time to suppress the 2D Zn^2+^ diffusion at the interface, leading to smooth Zn deposition [49, 50]. Moreover, the uniform Zn deposition can also be observed in the DMSO-based electrolyte (Fig. S2b), which is in good agreement with the previous report [51]. Therefore, it is reliable to employ DHM to investigate the interface evolution of the Zn anode during the initial deposition stage and further predict the possibility of dendrite growth at the subsequent stage.

The reverse Zn dissolution is also an important interfacial reaction of Zn anode, because it determines the initial surface configuration of the subsequent deposition process [52]. Herein, Zn dissolution in blank ZnSO_4_/H_2_O and ZnSO_4_/H_2_O/DMSO electrolyte at the current density of 1 mA cm^−2^ is in situ observed by DHM. The increased polarization (Fig. S2a) may be due to the decreased ion conductivity (Fig. S3) and increased viscosity of the electrolyte (Fig. S4) with the addition of DMSO. As shown in Fig. S2c, the uneven increase of the ion concentration appears at the interface after only 20 s in the blank electrolyte, indicating that the uneven Zn striping starts from a quite early dissolution stage in the blank electrolyte. The phenomenon becomes more and more obvious in the follow-up stage, which leads to the localized accelerated dissolution of Zn anode and result in a rough surface with obvious cracks (Fig. S5a). After striping for 1 h, the regional uneven stripping becomes serious (Fig. S5b). In contrast, uniform interfacial ion distribution is obtained in ZnSO_4_/H_2_O/DMSO electrolyte and no obvious ion accumulation is observed even after 60 s, indicating a uniform Zn dissolution with the presences of DMSO. It should be noted that the diffusion layer is becoming thicker with the presence of DMSO, due to the slowed ion transportation at the interface. The uniform Zn dissolution in ZnSO_4_/H_2_O/DMSO electrolyte provides a smooth surface for the Zn deposition that follows and effectively depresses the growth of dendrites, which can be confirmed further by the SEM images of the Zn anode after striping for 90 s and 1 h (Fig. S5c, d).

The results presented in the previous section prove that DHM can capture direct information of the electrolyte phase and provide insights into the mechanisms of numerous electrochemical processes occurring on the electrode/electrolyte interface. Possessing the advantages of simple operation and fast response, DHM is an ideal technique for screening electrolyte additives.

### Early-Stage Observation of the Zn Anode in TAA-based Electrolyte

**Fig. 2:**
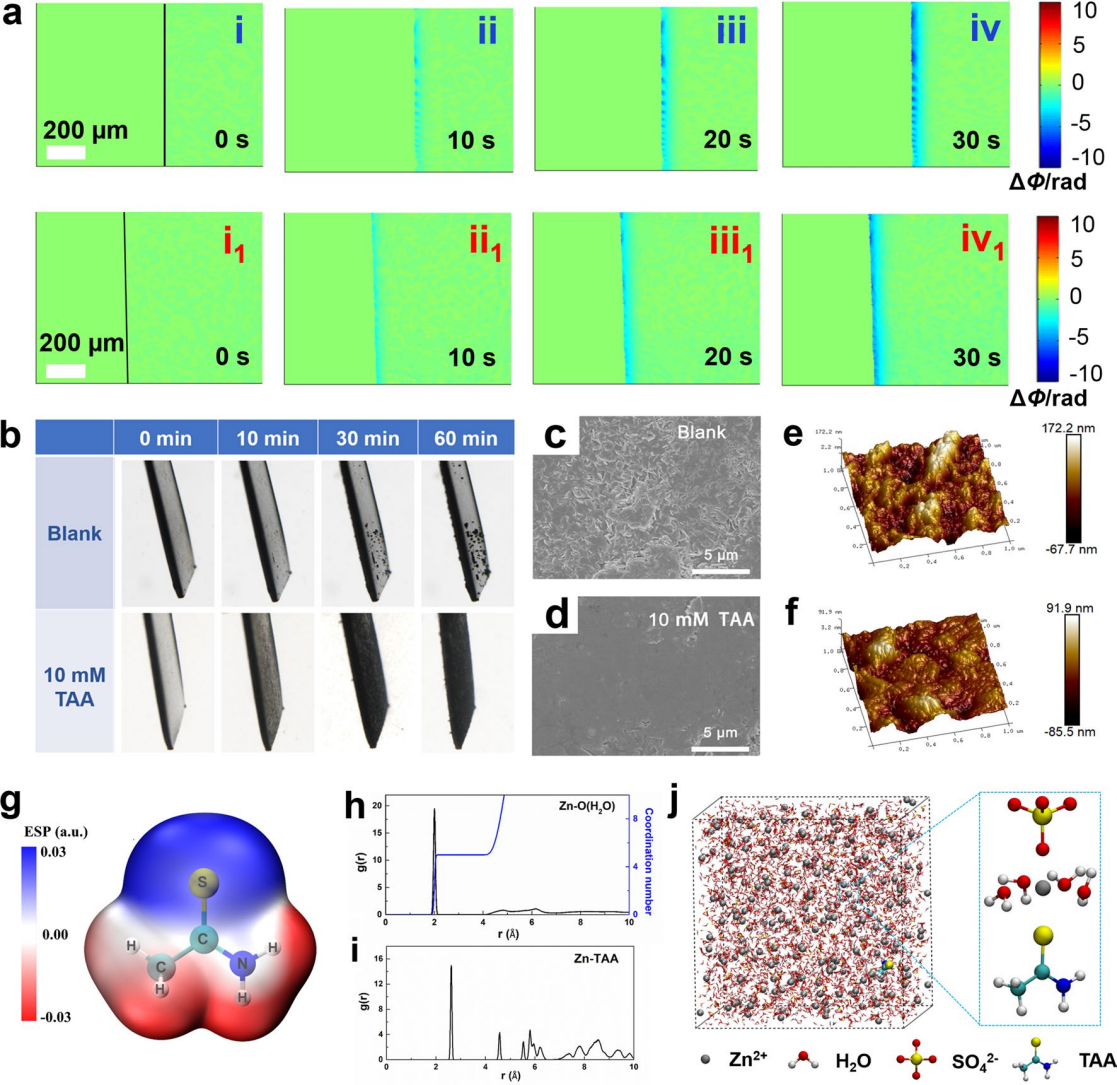
**a** The phase maps corresponding to different Zn deposition time at the current density of 1.0 mA cm^−2^ in (i–iv) blank ZnSO_4_ and (i_1_–iv_1_) ZnSO_4_-10 mM TAA electrolytes. **b** In situ optical microscopic observation of Zn deposition at 1 mA cm^−2^ in blank ZnSO_4_ and ZnSO_4_-10 mM TAA electrolytes. SEM images and AFM images of the Zn anodes after 3 cycles: **c, e** in blank ZnSO_4_, **d, f** in ZnSO_4_-10 mM TAA electrolytes. **g** Electrostatic potential mapping of the TAA molecule. The radial distribution functions of **h** Zn–O (H_2_O) and **i** Zn-TAA and their radius-dependent coordination numbers in ZnSO_4_-10 mM TAA electrolyte. **j** Snapshot of the MD simulation cells for TAA-based electrolyte

TAA possesses strong complexation ability with metal ions and is usually applied as a bath additive to improve the coating structure [53]. So, the effect of TAA on Zn deposition in 2 M ZnSO_4_ electrolyte is evaluated by DHM. As shown in Fig. 2a, uneven ion consumption emerges at the deposition time of 10 s at the current density of 1.0 mA cm^−2^, and becomes more and more serious in the subsequent deposition process. With the addition of TAA, uniform ion concentration gradient is formed within the initial 30 s, and remains stable thereafter, indicating that uniform deposition can be achieved with TAA electrolyte additive. As shown in Fig. S6, at the higher current density of 5.0 mA cm^−2^, heterogeneous ion concentration gradient over a large area is obviously observed in blank electrolyte, indicating that uneven Zn deposition occurs at the very early stage. With the increase of deposition time, regional ion concentration distribution becomes more and more obvious at the interface. In contrast, uniform diffusion layer is detected during the initial deposition process and it keeps stable in the following process in the electrolyte with TAA.

To confirm our inference, the surface evolution of the Zn anode during the initial deposition process was in situ observed with an optical microscope. As shown in Fig. 2b, uneven Zn deposition is observed after 10 min in the blank electrolyte, which becomes more and more serious in the follow-up process due to the “tip effect”. While even Zn distribution without obvious sediment accumulation is observed with the presence of TAA, indicating that TAA electrolyte additive can effectively inhibit dendrite growth. The corresponding SEM images shown in Fig. S7 further confirm that with the increase of deposition time, messy sheet-like dendrites gradually grow up and cracks are obviously observed in blank electrolyte, while a smooth and dense surface without obvious accumulation is obtained with the present of TAA additive. The SEM images (Fig. 2c, d) and atomic force microscopy (AFM, Fig. 2e, f) images of the Zn andoe after 3 cycles further demonstrate that a flat surface with the roughness of 18.1 nm is achieved in the presence of TAA electrolyte additive, which is more smooth than that obtained in blank electrolyte (roughness is 37.5 nm). Above results experimentally confirm that TAA electrolyte additive could realize uniform Zn deposition during the charge–discharge process, which are in good agreement with the DHM prediction.

### Effect Mechanism of TAA on Uniform Zn Deposition

**Fig. 3:**
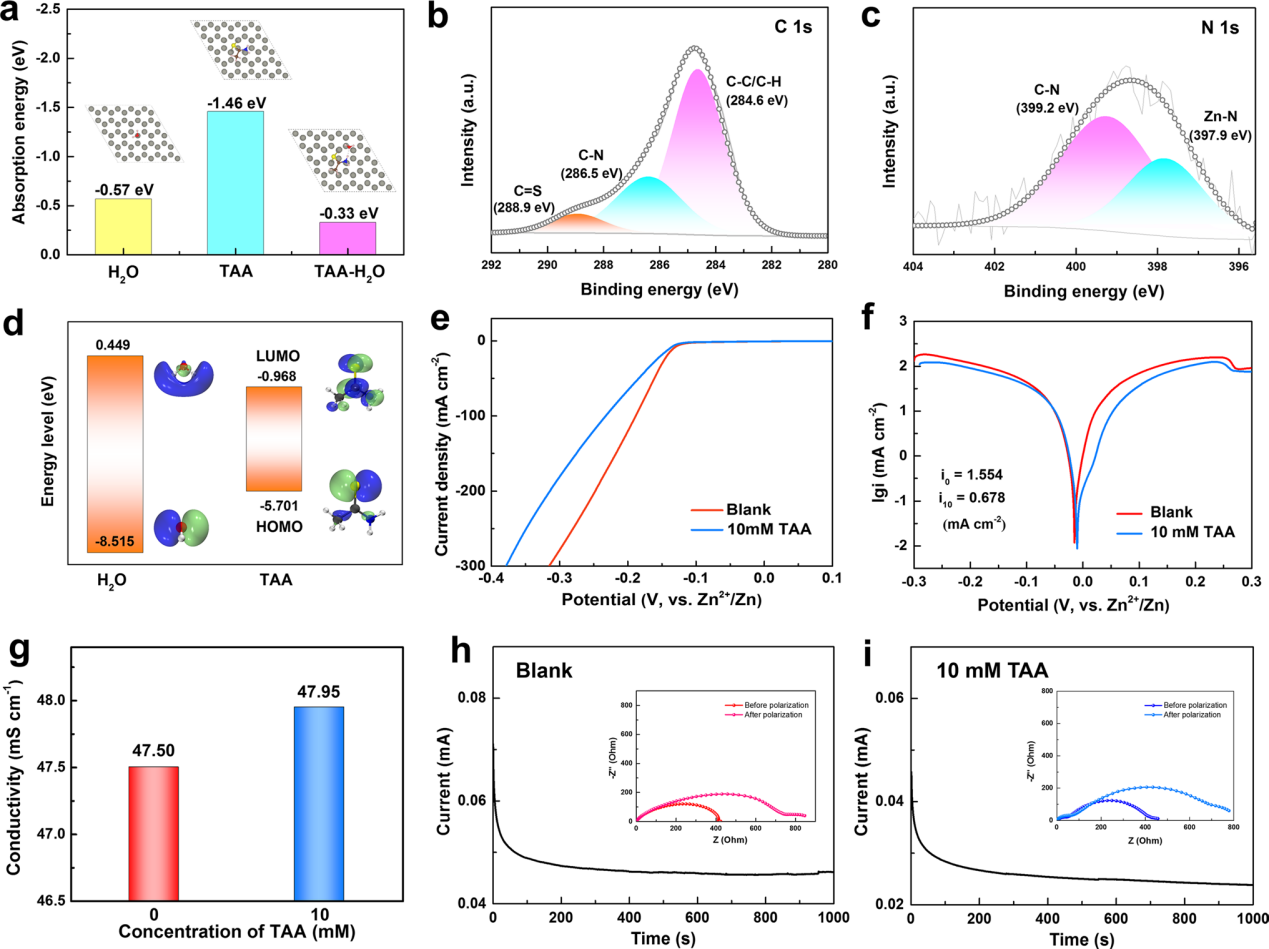
**a** Adsorption energies of H_2_O and TAA on Zn (002) surface, and adsorption energy of H_2_O on Zn (002) surface adsorbed TAA. High-resolution XPS spectra: **b** C 1*s*, and** c** N 1*s* for the Zn electrode after 3 cycles in ZnSO_4_-10 mM TAA electrolyte. **d** LUMO and HOMO isosurfaces of H_2_O molecules and TAA molecules. **e** The linear sweep voltammetry curves and **f** linear polarization curves of Zn anode obtained in the electrolytes with/without TAA additive. **g** Ionic conductivities of the electrolytes with/without TAA. Nyquist plots of the Zn-Zn symmetric cells before and after CA test and the corresponding CA curves obtained at an overpotential of 25 mV in the electrolytes: **h** blank ZnSO_4_, **i** ZnSO_4_-10 mM TAA electrolyte

To reveal the effect mechanism of TAA on uniform Zn deposition, DFT calculations were conducted. The van der Waals potential diagram of a TAA molecule shown in Fig. 2g displays that the nitrogen atom in TAA possesses the most negative electrostatic potential (ESP) value, indicating the concentration of negative charge and strong zincophilic affinity of this site [54]. It is inferred that the solvation configuration of hydrated zinc ion could be easily changed by the coordination of TAA via the lone pair electrons of nitrogen atom with Zn^2+^. Therefore, the molecular dynamics (MD) simulation is used to investigate the slovation structure of Zn^2+^ in the electrolytes without/with TAA additive. As shown in Fig. S8a, b, in the blank ZnSO_4_ electrolyte, sharp peaks of Zn–O (H_2_O) and Zn–SO_4_ appear at 0.2 and 0.22 nm, respectively, in the radial distribution functions (RDFs) graph. The coordination number (CN) analysis further confirms that Zn^2+^ is surrounded by five H_2_O and one SO_4_ at the primary solvation shell, corresponding to the MD simulation shown in Fig. S8c. In comparison, with the presence of TAA, a RDF peak of Zn-TAA is located at ~ 0.26 nm, demonstrating the involvement of TAA molecules in the first solvation structure of Zn^2+^ (Fig. 2h–j). The nuclear magnetism (NMR) measurements shown in Fig. S9 present an obvious shift of ^1^H peak with the presence of TAA additive, experimentally confirming the destruction of hydrogen-bond network in ZnSO_4_ electrolyte. The change of the coordination environment of Zn^2+^ is favorable for regulating the interficial ion flux and inhibiting the activity of free water at the interface. The effect of TAA electrolyte additive on the electrode/electrolyte interface is also investigated by DFT method. As shown in Fig. 3a, TAA molecues shows the adsorption energy of −1.46 eV on the Zn (002) surface, which is lower than that of −0.57 eV for H_2_O molecules, indicating that a TAA adsorption layer is preferably formed on Zn surface. Noteably, after TAA adsorption, H_2_O displays greatly weakened adsorption ability on Zn surface (adsortion energy is −0.33 eV), implying that TAA adsorption layer possesses a specific waterproof property. Therefore, it is reasonable to deduce that with the addition of TAA, a stable interface adsorption layer can be constructed on the surface of Zn anode and protect it from chemical corrosion caused by the direct contact with electrolyte. XPS survey spectra (Fig. S10) and EDS mapping (Fig. S11) of the Zn anode after 3 cycles show obvious existence of Zn, C, O, S and N elements on the surface. The characteristic peaks of C=S, C–C/C–H and C–N bonds detected in the C 1*s* spectrum (Fig. 3b) prove the adsorption of TAA on the surface of the electrode. The Zn-N peak appearing at 397.7 eV in the N 1*s* spectrum (Fig. 3c) further confirms that N atom is chemically bonded to Zn atom site.

The physical properties of electrolyte is easily influenced by addtives, which could further impact the electrochemical performance of ZIBs. To investigate the effect of TAA on the electrochemical stabililty of the electrolyte, the highest occupied molecular orbital (HOMO) and lowest unoccupied molecular orbital (LUMO) for the H_2_O solvent and TAA additive molecules are studied by DFT calculation. As shown in Fig. 3d, the TAA additive delivers far lower LUMO (−0.968 eV) than the H_2_O (0.449 eV), indicating that TAA is more preferentially reduced than H_2_O and could be able to resrain the deposition of the H_2_O solvent. The linear sweep voltammetry (LSV) method is used to evaluate the effect of TAA on the hydrogen evolution reaction (HER) of the electrolyte. As shown in Fig. 3e, the onset potential of HER shifts negatively with the introduction of TAA, demonstrating that the hydrogen evolution is significantly inhibited, which is in good agreement with the theoretical calculation results. The anti-corrosion performance of the Zn anode is further evaluated by Tafel polarization curves. As shown in Fig. 3f, the Zn anode delivers lower exchange current density of 0.678 mA cm^−2^ in the electrolyte with 10 mM TAA additive than that of 1.554 mA cm^−2^ obtained in blank ZnSO_4_ electrolyte, implying the effectively reduced side-reactions during the Zn deposition/dissolution process. Moreover, the morphology characterization of the Zn foil after being immersed in each electrolyte for 6 h (Fig. S12) further confirms that TAA helps to suppress the corrosion of Zn foil and avoid the formation of by-products, which could release the passivation of Zn anode. Ionic conductivity measurements of the electrolytes shown in Fig. 3g reveal a faster Zn^2+^ migration in the electrolyte with TAA additive, which could contribute to the deposition/dissolution dynamics. Moreover, according to the EIS and CA experiments shown in Fig. 3h, i, the Zn^2+^ transfer numbers in blank ZnSO_4_ and ZnSO_4_-10 mM TAA electrolytes are calculated to be 0.374 and 0.602, respectively, further demonstrating the accelerated Zn^2+^ ion mobility rate with the presence of TAA. The enhanced ion conductivity and higher ion transference number will contribute to reduce the interficial concentration gradient, and is expected to lower the polarization and induce homogeneous Zn deposition.

### Electrochemical Properties of the Symmetric Cells

**Fig. 4:**
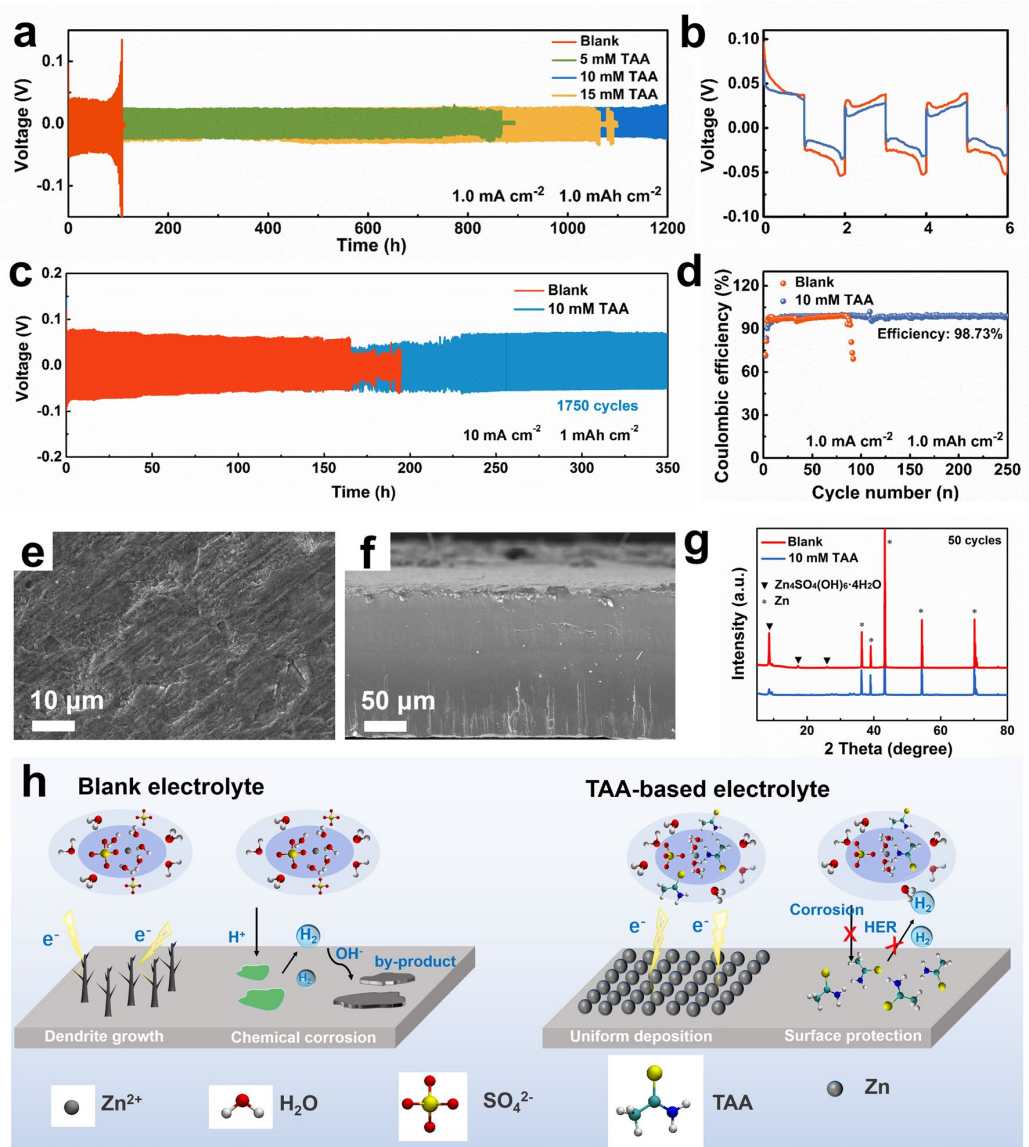
**a** Galvanostatic cycling property of Zn-Zn symmetric cells in the electrolytes with and without TAA at the current density of 1 mA cm^−2^ with the areal capacity of 1 mAh cm^−2^, and **b** the enlarged charge–discharge curves of the initial 3 cycles. **c** Cycling performance of the symmetric cells at high current density of 10 mA cm^−2^ with the areal capacity of 1 mAh cm^−2^. **d** Coulombic efficiency measurements of the Zn-Cu cells at a current density of 1 mA cm^−2^ with the areal capacity of 1 mAh cm^−2^. SEM images of the Zn anode after 50 cycles in ZnSO_4_-10 mM TAA electrolytes: **e** top view, **f** side view. **g** XRD patterns of the Zn anode after 50 cycles in the electrolytes. **h** Schematic illustration of effect mechanism of TAA on Zn deposition in electrode/electrolyte interface in blank ZnSO_4_ and ZnSO_4_-TAA electrolytes

To examine the function of TAA electrolyte additive during the long-term Zn plating/stripping, Zn–Zn symmetric batteries using the electrolytes with different concentration of TAA additive were assembled and tested. Figure 4a illustrates that the symmetric cell using ZnSO_4_-TAA electrolyte delivers superior cycling stability than that using blank electrolyte. When the concentration of TAA is 10 mM, the cell displays an outstanding cycle life of over 1200 h at the current density of 1 mA cm^−2^ with the areal capacity of 1 mAh cm^−2^. While that using blank ZnSO_4_ electrolyte is short-circuited after 108 h, which may be associated with the uncontrolled dendrite growth (Fig. S13). Enlarged voltage profiles of the initial 3 cycles (Fig. 4b) show that the polarization of the battery with TAA (~ 46 mV) is generally lower than that without TAA (~ 63 mV), implying lower energy barrier for Zn deposition with the presence of TAA. In addition, even at the higher current density of 2 mA cm^−2^ and higher areal capacity of 2 mAh cm^−2^ (Fig. S14), the Zn–Zn cell using TAA-based electrolyte still keeps stable for ~ 500 h. The rate performance measurements (Fig. S15) at various current densities from 1 to 4 mA cm^−2^ reveal as well that the cell using TAA-based electrolyte exhibits substantially lower voltage hysteresis than that using blank electrolyte, suggesting lower polarization and good kinetics with the presence of TAA. At the higher current density of 10 mA cm^−2^ with the areal capacity of 1 mAh cm^−2^, the Zn anode keeps stable after 1,750 cycles (Fig. 4c). It is probable that the addition of TAA reliefs the accumulation of detrimental by-products and unclogs the electron/ion transportation at the interface. The morphology of the Zn anode after 50 cycles in TAA based electrolyte has been characterized by SEM. As shown in Fig. 4e, f, the Zn anode exhibits smooth surface without obvious cracking or dendrites from both the top view and side view, confirming that dendrite-free Zn deposition can be realized by TAA additive even after long term cycling. The corresponding XRD patterns (Fig. 4g) demonstrate that strong peaks for Zn_4_(OH)_6_SO_4_·4H_2_O are observed in blank electrolyte, while no obvious peak for byproduct is observed in the TAA-based electrolyte, further indicating that chemical/electrochemical corrosion has been effectively inhibited by TAA additive.

CE is also tested in Zn//Cu cells to evaluate the Zn plating/stripping efficiency in the TAA-based electrolytes. As shown in Fig. 4d, after 80 cycles, the CE values of the Zn//Cu cell using ZnSO_4_ electrolyte start to fluctuate and then decrease in the follow-up cycles, which may be caused by the dendrite growth and side-reactions. In contrast, for the cell using ZnSO_4_-TAA electrolyte, its initial CE is close to that of using blank electrolyte, but it reaches a very high and stable plating/stripping efficiency of 98.73% within 10 cycles and keeps stable for over 250 cycles. The excellent stability and enhanced CE of Zn anode can be attributed to the construction of water-poor interface and accelerated ionic transmission, which not only alleviate the undesired side reactions but also accelerate the deposition dynamics of Zn^2+^.

Based on the above discussions, the enhanced electrochemical performance of Zn anode in TAA-based electrolyte should be ascribed to the mechanism illustrated in Fig. 4h. On one hand, the solvation configuration change of hydrated Zn^2+^ accelerates the ion transportation in the electrolyte and balances the ion flux at interface during the charge/discharge process, thus significantly inhibits the “tip effect” commonly occurring in blank ZnSO_4_ electrolyte and induces homoneous Zn deposition without dendrite growth. On the other hand, the adsorption of TAA molecules on the surface of Zn anode constructs a water-proof interface, thus suppresses the chemical corrosion and prevent the surface passivation caused by the direct contact of electrolyte with Zn anode. The bifunctions of TAA additive endow Zn anode with excellent stability and high CE during the long-term plating/striping process, which is in good agreement with the prediction of the DHM results, thus confirming that DHM is a simple yet effective technology in screening electrolyte additives.

### Electrochemical Performance of the Zn–V_2_O_5_ Batteries

**Fig. 5:**
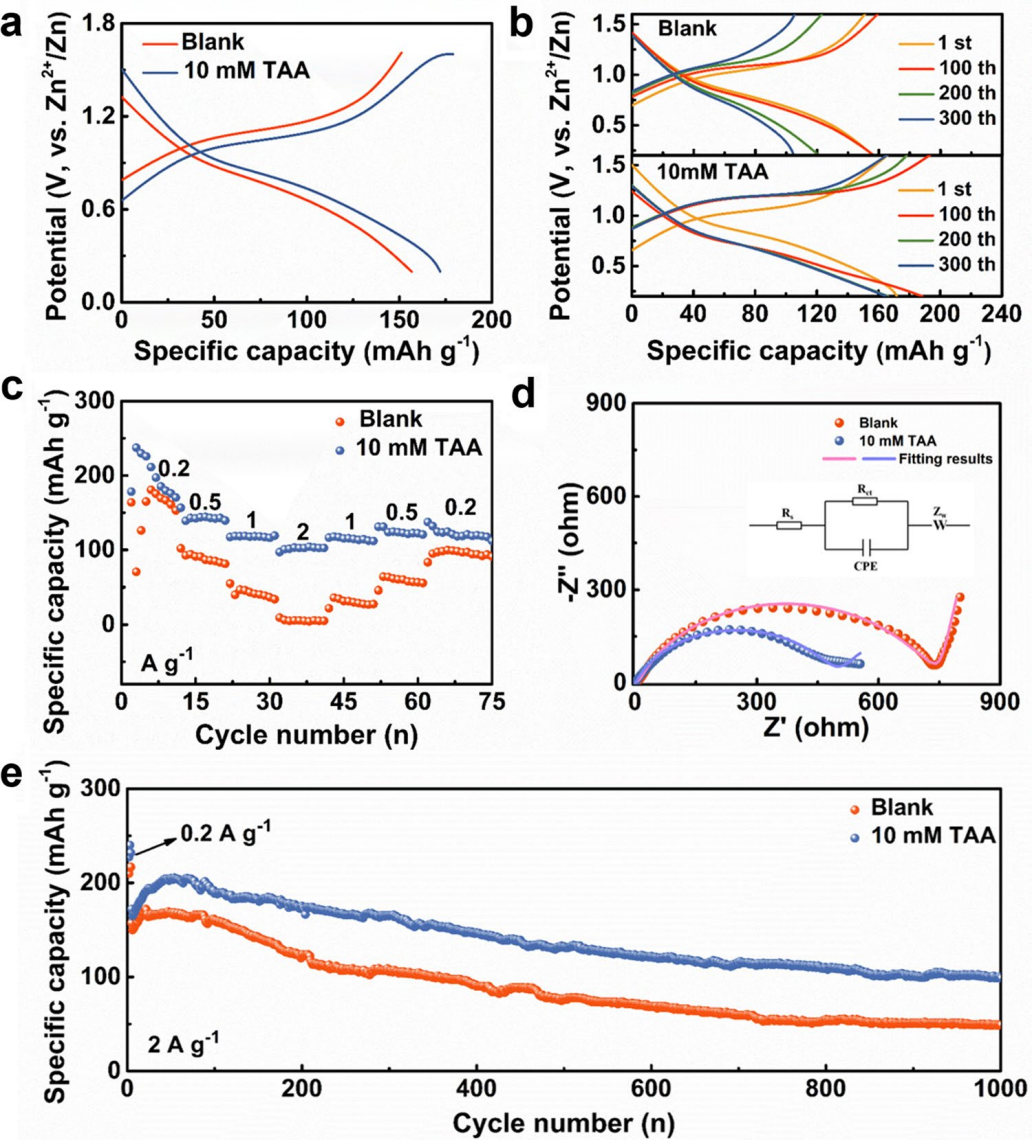
Performance comparison of Zn-V_2_O_5_ full cells between 2 M ZnSO_4_ and 2 M ZnSO_4_-10 mM TAA electrolytes. **a** GCD curves under a current density of 2 A g^−1^. **b** GCD curves of different cycling times. **c** Rate capacities under different current densities ranging from 0.2 to 2 A g^−1^. **d** Nyquist plots and the fitting results obtained in 2 M ZnSO_4_ and 2 M ZnSO_4_-10 mM TAA electrolytes (insertion is the equivalent circuit). **e** Cycling stability of the full cells under a current density of 2 A g^−1^

To emphasize the positive influence of TAA electrolyte additive in the practical application of ZIBs, V_2_O_5_ was employed as the cathode material for full batteries. Clearly, in the electrolyte with a small amount of TAA, the overall performance of Zn-V_2_O_5_ cells is significantly enhanced. The galvanostatic charge–discharge (GCD) curves in Fig. 5a, b show that in the TAA-based electrolyte, the cells display reduced polarization and higher initial discharge capacity of 172.6 mAh g^−1^, as well as superior cycling stability compared with those in blank electrolytes. Figure 5c shows that when the current density is gradually increased from 0.2 to 2.0 A g^−1^, the cells in TAA-based electrolyte exhibit enhanced rate performance, which may be attributed to the reduced charge transfer resistance from 785 Ω for blank electrolyte to 476 Ω for TAA-based electrolyte (Fig. 5d). The cycling performance of the full cells are further evaluated at the current density of 2.0 A g^−1^. As shown in Fig. 5e, after an active process (the first 3 cycles at the current density of 0.2 A g^−1^), the Zn–V_2_O_5_ full cell using ZnSO_4_-TAA electrolyte delivers a much higher specific capacity and better cycle stability. The specific capacity maintains 98.8 mAh g^−1^ after 1000 cycles.

## Conclusions

In summary, based on the in situ detection of the changes at the electrolyte side of the electrode/electrolyte interface with DHM, the phase maps obtained in blank electrolyte at various current densities and in DMSO-based electrolyte prove that DHM is ideal for the study of the interfacial dynamic evolution of Zn anode. Due to its high temporal resolution, DHM excels in the detection of non-uniform reactions taking place at the electrode/electrolyte interface, especially the changes of the liquid phase during the initial deposition/dissolution process. In addition, uniform Zn dissolution, as well as its deposition, can be observed with DHM. Further investigations into the TAA-based electrolyte system prove that it is effective, quick and convenient in the screening of electrolyte additives according to holographic measurements. Mechanism investigations reveal that the TAA additive could change the ion complex environment and form a water-poor layer, which are helpful to regulate the interficial ion flux, thus inhibiting dendrite growth and side reactions. This work offers a new perspective for inspecting the growth of dendrites at the early deposition stage and for screening electrolyte additives for secondary batteries.

## Supplementary Information

Below is the link to the electronic supplementary material.

Supplementary file1 (MP4 693 kb)

Supplementary file2 (PDF 1398 kb)
