# Supplementary material for: Thioacetamide Additive Homogenizing Zn Deposition Revealed by In Situ Digital Holography for Advanced Zn Ion Batteries
Source: Nanomicro Lett. 2024 Feb 15;16:117. doi: 10.1007/s40820-023-01310-3 (PMC10869330; doi:10.1007/s40820-023-01310-3)
Supplement: Supplementary file 2 — Supplementary file2 (PDF 1398 kb) [file 40820_2023_1310_MOESM2_ESM.pdf]

Supporting Information for

Thioacetamide Additive Homogenizing Zn Deposition Revealed by *In Situ* Digital Holography for Advanced Zn Ion Batteries

Kaixin Ren[1, #], Min Li[1, #], Qinghong Wang[1, *], Baohua Liu[1], Chuang Sun[1], Boyu Yuan[2, *], Chao Lai[1], Lifang Jiao[3], and Chao Wang[1, *]

[1]School of Chemistry and Materials Science, Jiangsu Normal University, Xuzhou, Jiangsu 221116, P. R. China

[2]Jiangsu Key Laboratory of Advanced Laser Materials and Devices, School of, Physics and Electronic Engineering, Jiangsu Normal University, Xuzhou, Jiangsu 221116, P. R. China

[3]Key Laboratory of Advanced Energy Materials Chemistry (Ministry of Education), Nankai University, Tianjin 300071, P. R. China

[#] Kaixin Ren and Min Li contributed equally to this work

*Corresponding author. E-mail: wangqh@jsnu.edu.cn (Q. Wang), yuanby@jsnu.edu.cn (B. Yuan), wangc@jsnu.edu.cn (C. Wang)

S1 Details for DFT Calculations and MD Simulations

DFT simulations were conducted using CP2K and ORCA to calculate the adsorption energy and binding energy, respectively [S1]. Adsorption calculations were carried out using the CP2K package version-2022.1 using Gaussian Plane Wave (GPW) method implemented in the QUICKSTEP module [S2]. Perdew-Burke-Ernzerhof (PBE) [S3] exchange-correlation (XC) functional with Grimme-D3 [S4,S5] dispersion correction method was employed. Both the double-zeta valence polarized (DZVP) sets and Goedecker-Teter-Hutter (GTH) pseudopotentials were adopted [S6-S8]. Plane wave and relative cut-offs were set to 400 and 55 Ry, respectively. The inner and outer SCF convergence criteria were set to 2.0 × 10^{-6} Ha. The Zn foil was simulated using a four layers of Zn (0 0 2) slab model with 5 × 5 (39.97 Å × 39.97 Å) surface unit cell periodicity. In order to avoid interactions between periodic images, a vacuum distance of 20 Å was imposed between different layers. The geometrical optimizations were implemented at the Γ point for all surface structures. The bottom two layers of atoms were frozen while the top two were allowed to relax. Root mean square and maximum force convergence were set to 3.0 × 10^{-4} and 4.5 × 10^{-4} Ha·Å^{-1}, respectively.

The adsorption energies were calculated according to Eq. (S1),

$$E_{ads} = E_{(slab + adsorbate)} - E_{(slab)} - E_{(adsorbate)} \quad (S1)$$

where $E_{(slab + adsorbate)}$, $E_{(slab)}$, and $E_{(adsorbate)}$ are the calculated electronic energy of species adsorbed on the surface, the bare surface, and the gas-phase molecule, respectively.

Binding energy calculations were carried out using ORCA (5.0.3). Geometry optimization was conducted at the B97-3c level of theory [S9] The single point calculations for the optimized geometries were performed to obtain accurate energies at the ωB97X-V/def2-TZVP level of theory [S10-S12]. The solvent effect of water evaluated by the CPCM solvation model. The RIJCOSX approximation was applied with the def2/J auxiliary basis set [S13, S14].

The binding energy can be calculated by Eq. (S2), where AB represents the total energy after the binding between Zn^{2+} and the organic molecule. A and B are the energy of a single Zn^{2+} and organic molecule, respectively.

$$E_{binding} = E(AB) - E(A) - E(B) \qquad (S2)$$

MD simulations were conducted using GROMACS software package (2020.6 version). The gromos54a7 force field were used to parametrize all atoms, such as the bond parameters, angle parameters and the dihedral angles, and so on. The RESP charge of SO_4^{2-} and TAA molecule was calculated using ORCA at the B3LYP/6-311+g(d,p) level. TIP3P was used for the model of water molecule. The steepest descent method was applied to minimize the initial energy for each system with a force tolerance of 1 kJ/(mol^{-1} nm^{-1}) and a maximum step size of 0.002 ps before MD calculations. In all the three directions, periodic boundary conditions were imposed. Leapfrog algorithm was used to integrate the Newtonian equation of motion. The MD simulation was processed in an NPT ensemble and the simulation time is 20 ns. In NPT simulations, the pressure was maintained at 1 bar by the Berendsen barostat in an isotropic manner was performed for constrain bond lengths of hydrogen atoms. The Particle-Mesh-Ewald (PME) with a fourth-order interpolation was used to evaluate the electrostatic interactions and the grid spacing is 1.0 Å, whereas a cutoff of 1.0 Å was employed to calculate the short-range van der Waals interactions. After 20 ns of MD simulations, the radial distribution function (RDF) and coordination number between Zn^{2+} and H_2O, SO_4^{2-}, TAA molecules were calculated [S15].

Supplementary Figures and Tables

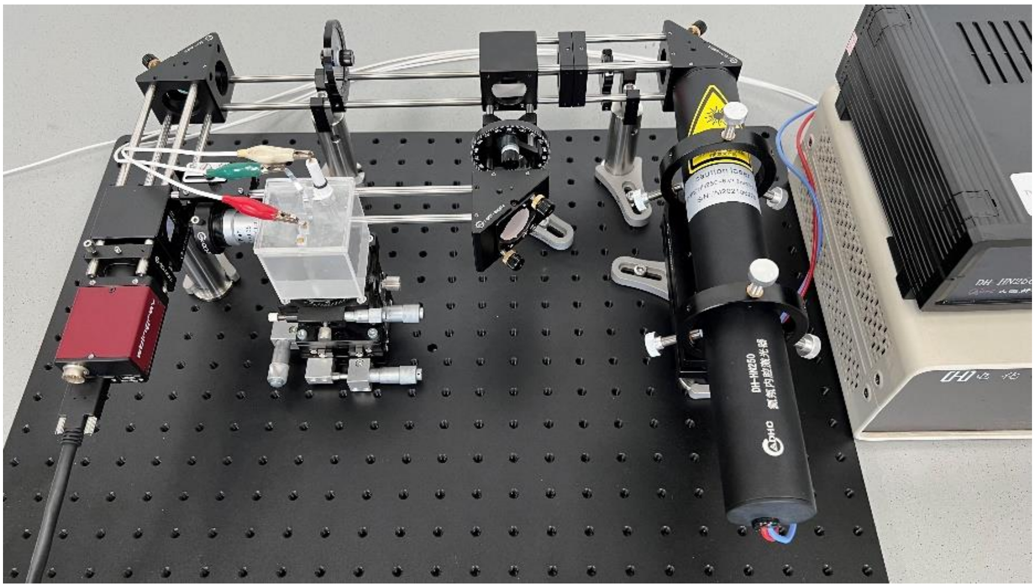

Fig. S1 The photo of DHM equipment

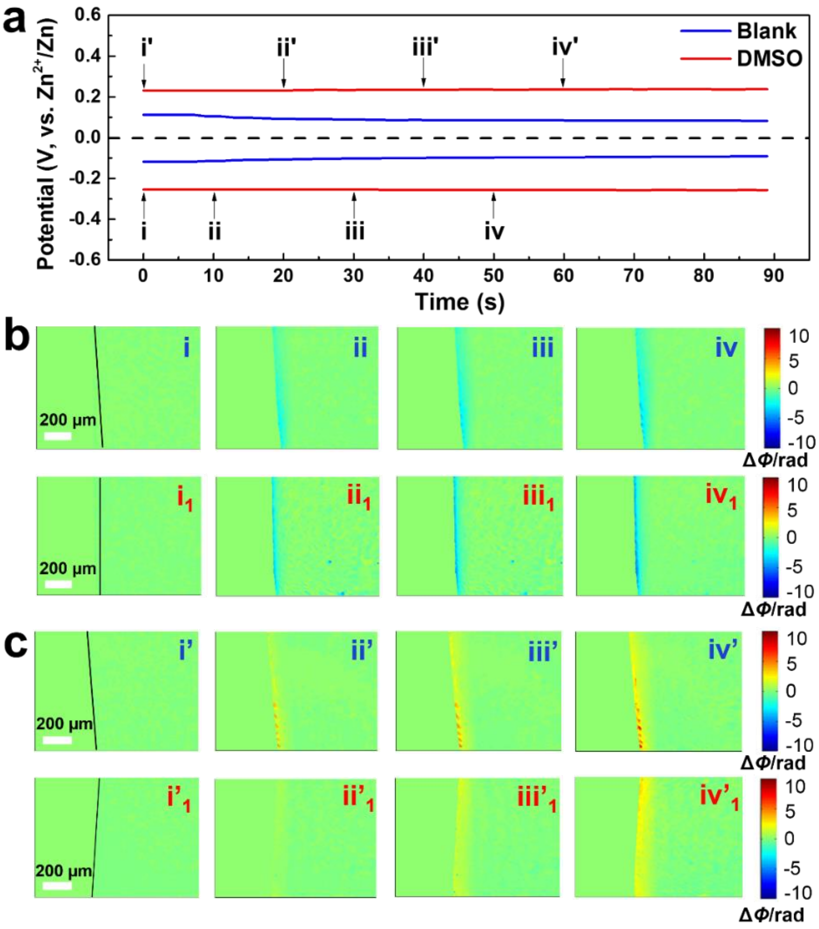

Fig. S2 a The voltage profiles of galvanostatic Zn plating and striping at the current density of 1.0 mA cm^{-2} in 0.5 M $ZnSO_4$ blank and DMSO-based electrolyte. **b** The phase maps corresponding to points (i-iv) in (a). i-iv: $ZnSO_4$ blank electrolyte, i1-iv1: $ZnSO_4$ DMSO-based electrolyte. **c** The phase maps corresponding to points (i'-iv') in (a). i'-iv': $ZnSO_4$ blank electrolyte, i'$_1$-iv'$_1$: $ZnSO_4$ DMSO-based electrolyte

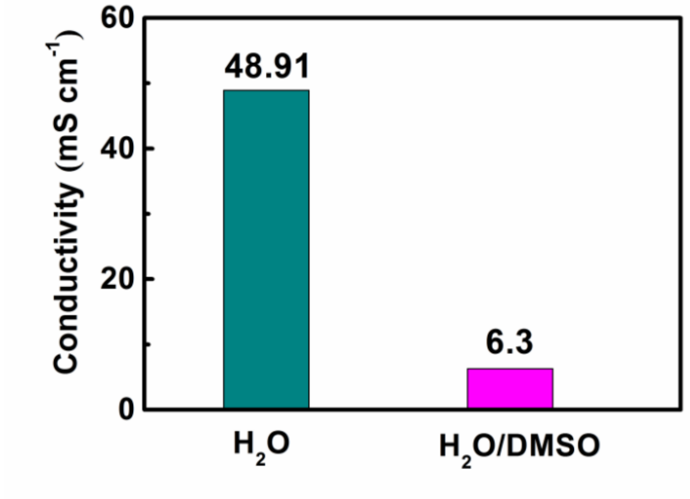

Fig. S3 Ion conductivity of the 2 M $ZnSO_4$ electrolytes in H_2O and H_2O/DMSO (volume ration=1:1) system

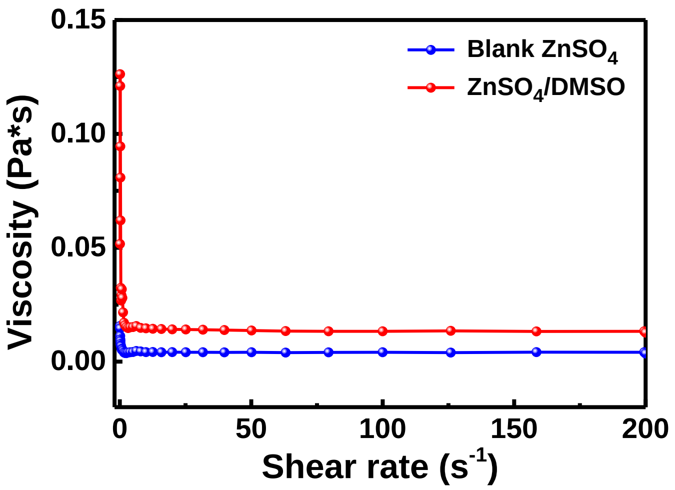

Fig. S4 Viscosity of the 0.5 M $ZnSO_4$ electrolyte with and without DMSO

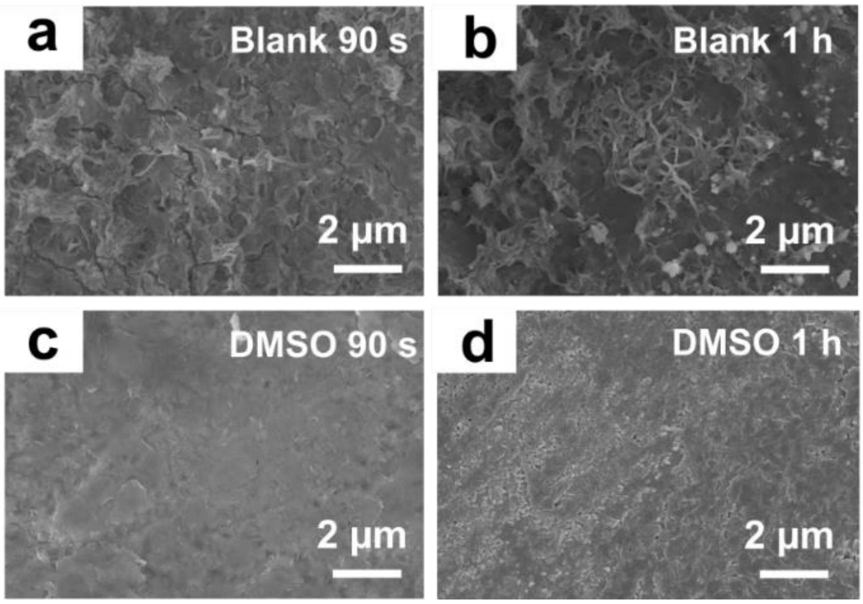

Fig. S5 SEM images of the Zn foil after 90 s and 1 h dissolution at the current density of 1 mA cm^{-2} in the electrolytes: **a, b** blank 0.5 M $ZnSO_4$ electrolyte, **c, d** DMSO-based electrolyte

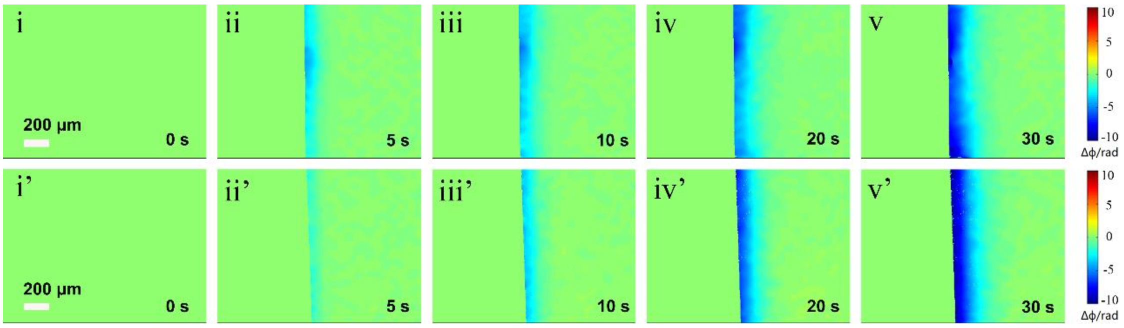

Fig. S6 The phase maps corresponding to different Zn deposition time at the current density of 5 mA cm^{-2} in (i-iv) blank $ZnSO_4$ and (i'-iv') $ZnSO_4$-10 mM TAA electrolytes

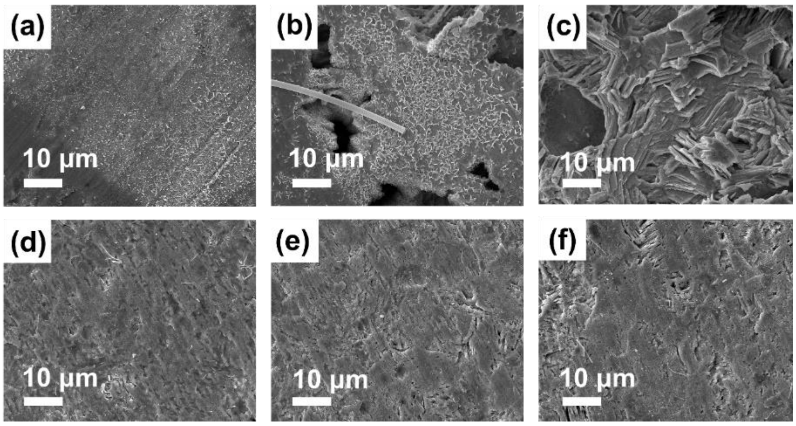

Fig. S7 SEM images of the Zn anode after 10 min, 30 min and 60 min deposition in (**a-c**) blank electrolyte and (**d-f**) TAA added electrolyte

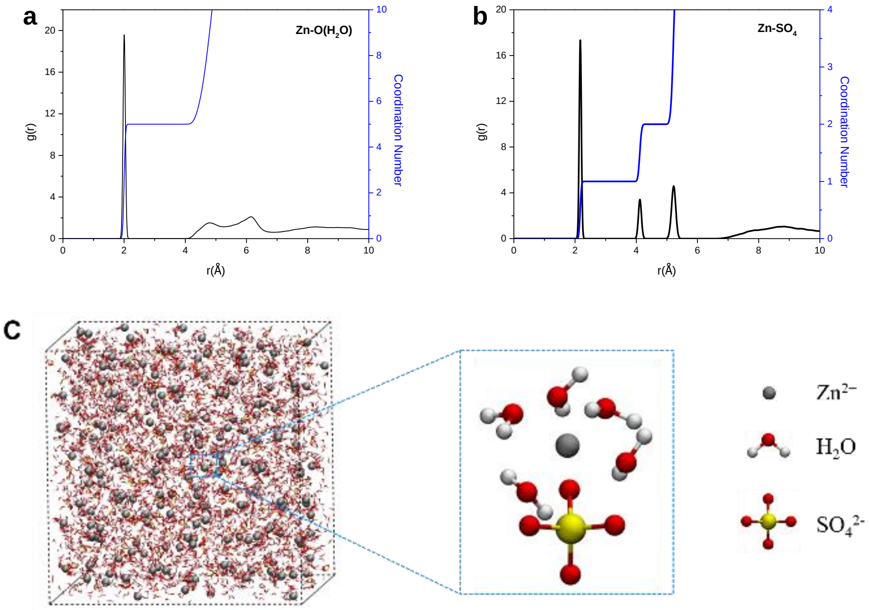

Fig. S8 The radial distribution functions of (**a**) Zn-O(H_2O) and (**b**) Zn-SO_4^{2-} and their radius-dependent coordination numbers in blank $ZnSO_4$ electrolyte. (**c**) Snapshot of the MD simulation cells for blank $ZnSO_4$ electrolyte

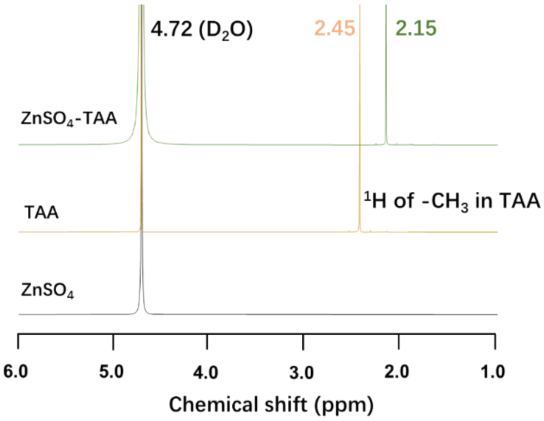

Fig. S9 Comparison of the ^{1}H NMR spectra of the solution containing $ZnSO_4$, TAA, and mixture of $ZnSO_4$ with TAA in D_2O

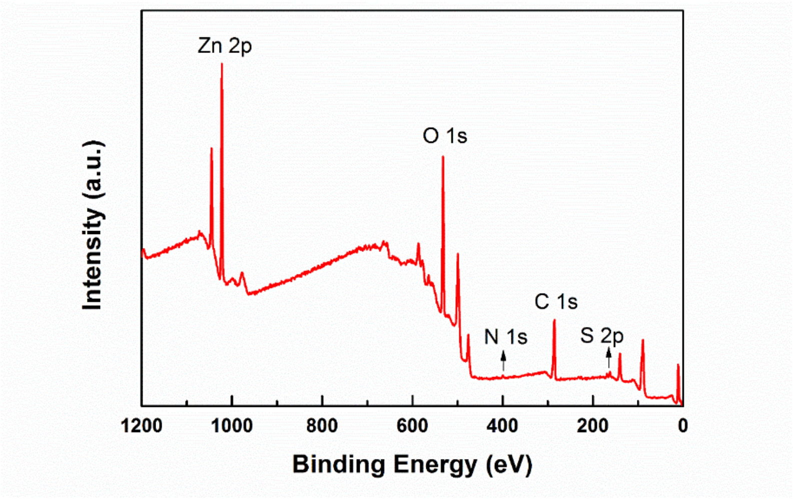

Fig. S10 XPS survey spectra of the Zn electrode after 3 cycles in $ZnSO_4$-10 mM TAA electrolyte

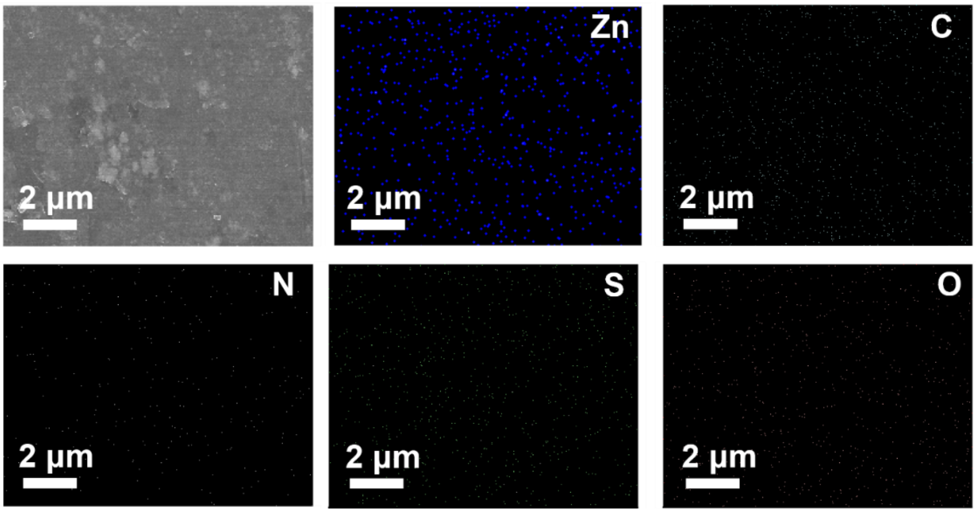

Fig. S11 SEM image and the corresponding Zn, C, N, S and O mapping of the Zn anode after 3 cycles in $ZnSO_4$-10 mM TAA electrolyte

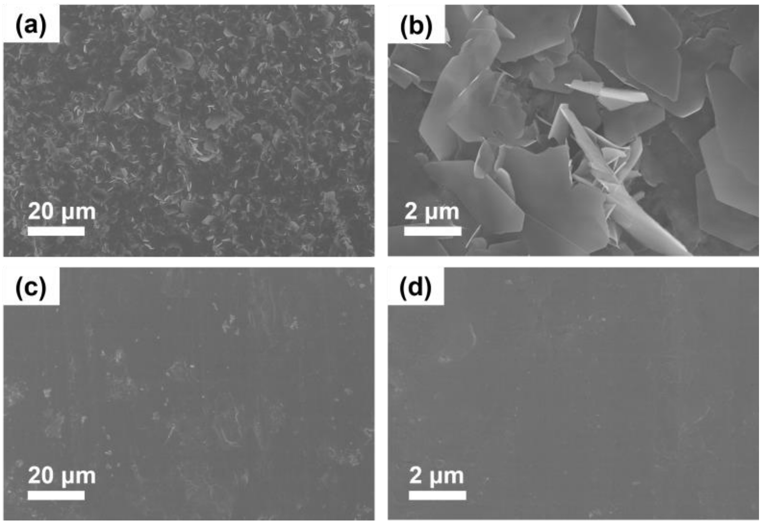

Fig. S12 SEM images of the Zn foils after immersing in the electrolytes with and without TAA for 6 h: (**a, b**) blank ZSO electrolyte, (**c, d**) TAA-based electrolyte

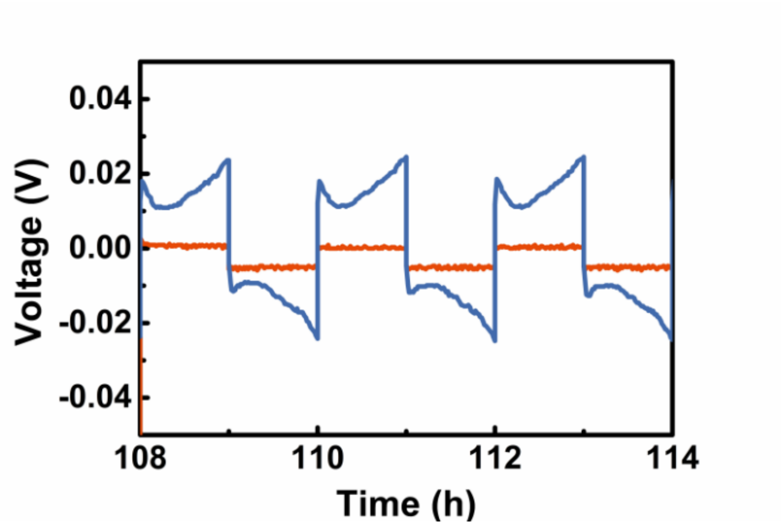

Fig. S13 Enlarged galvanostatic charge-discharge curves of the symmetric cells with and without TAA at the deposition time of 108~114 h

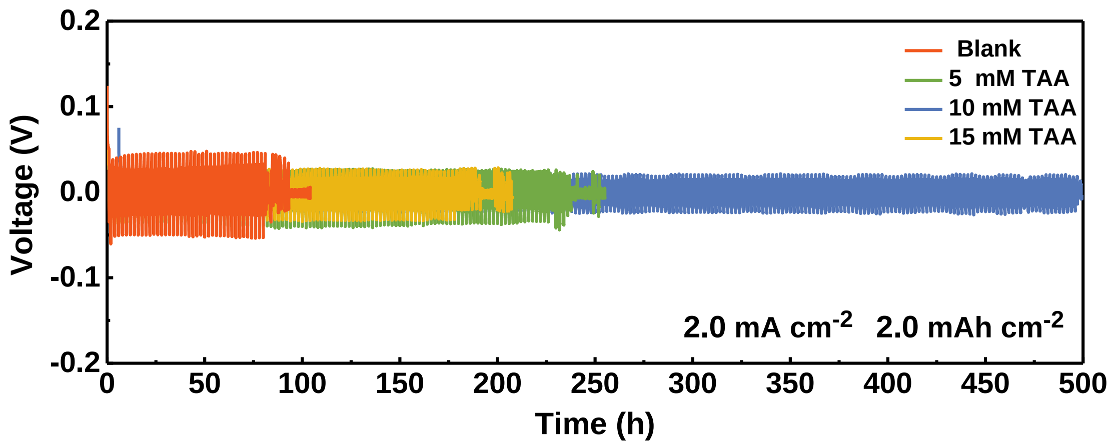

Fig. S14 Voltage-time profiles of the cells using TAA-based electrolytes at the current density of 2 mA cm^{-2} with the capacity of 2 mAh cm^{-2}

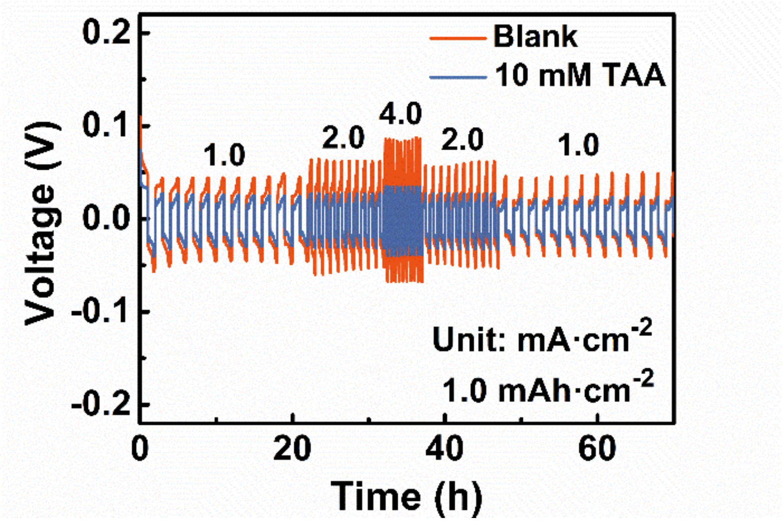

Fig. S15 Rate performance of the Zn-Zn cells with the current density steply increased from 1.0 to 4.0 A cm^{-2} and decreased to 1.0 A cm^{-2}

Movie S1 a The voltage profile of galvanostatic Zn deposition at the current density of 1.0 mA cm^{-2} in 2.0 M $ZnSO_4$ electrolytes; **b** the corresponding dynamic evolution of interference fringes in the initial 40 s; **c** the corresponding dynamic evolution of phase maps in the initial 40 s.

Supplementary References

[S1] X.R. Gan, J. Tang, X.Y. Wang, L. Gong, I. Zhitomirsky et al., Aromatic additives with designed functions ameliorating chemo-mechanical reliability for zinc-ion batteries. Energy Stor. Mater. **59**, 102769 (2023). https://doi.org/10.1016/j.ensm.2023.102769

[S2] J. VandeVondele, M. Krack, F. Mohamed, M. Parrinello, T. Chassaing et al., Quickstep: Fast and accurate density functional calculations using a mixed Gaussian and plane waves approach. Comput. Phys. Comm. **167**, 103-128 (2005). https://doi.org/10.1016/j.cpc.2004.12.014

[S3] J.P. Perdew, K. Burke, M. Ernzerhof, Generalized Gradient Approximation Made Simple. Phys. Rev. Lett. **77**, 3865-3868 (1996). https://doi.org/10.1103/PhysRevLett.77.3865

[S4] S. Grimme, J. Antony, S. Ehrlich, H. Krieg, A consistent and accurate ab initio parametrization of density functional dispersion correction (DFT-D) for the 94 elements H-Pu. J. Chem. Phys. **132**, 154104 (2010). https://doi.org/10.1063/1.3382344

[S5] S. Grimme, S. Ehrlich, L. Goerigk, Effect of the damping function in dispersion corrected density functional theory. J. Comput. Chem. **32**, 1456-1465 (2011). https://doi.org/10.1002/jcc.21759

[S6] J. VandeVondele, J. Hutter, Gaussian basis sets for accurate calculations on molecular systems in gas and condensed phases. J. Chem. Phys. **127**, 114105 (2007). https://doi.org/10.1063/1.2770708

[S7] S. Goedecker, M. Teter, J. Hutter, Separable dual-space gaussian pseudopotentials. Phys. Rev. B **54**, 1703-1710 (1996). https://doi.org/10.1103/PhysRevB.54.1703

[S8] C. Hartwigsen, S. Goedecker, J. Hutter, Relativistic separable dual-space gaussian pseudopotentials from h to rn. Phys. Rev. B **58**, 3641-3462 (1998). https://doi.org/10.1103/PhysRevB.58.3641

[S9] J.G. Brandenburg, C. Bannwarth, A. Hansen, S. Grimme, B97-3c: A revised low-cost variant of the B97-D density functional method. J. Chem. Phys. **148**, 064104 (2018). https://doi.org/10.1063/1.5012601

[S10] N. Mardirossian, M. Head-Gordon, ωB97X-V: A 10-parameter, range-separated hybrid, generalized gradient approximation density functional with nonlocal correlation, designed by a survival-of-the-fittest strategy. Phys. Chem. Chem. Phys. **16**, 9904-9924 (2014). https://doi.org/10.1039/C3CP54374A

[S11] F. Weigend, R. Ahlrichs, Balanced basis sets of split valence, triple zeta valence and quadruple zeta valence quality for H to Rn: Design and assessment of accuracy. Phys. Chem. Chem. Phys. **7**, 3297 (2005). https://doi.org/10.1039/B508541A

[S12] A. Mirzanejad, S.A. Varganov, The role of the intermediate triplet state in iron-catalyzed multi-state C–H activation. Phys. Chem. Chem. Phys. **24**, 20721-20727 (2022). https://doi.org/10.1039/D2CP02733J

[S13] F. Neese, F. Wennmohs, A. Hansen, U. Becker, Efficient, approximate and parallel Hartree–Fock and hybrid DFT calculations. A ‘chain-of-spheres’ algorithm for the

Hartree–Fock exchange. Chem. Phys. **356**, 98-109 (2009). https://doi.org/10.1016/j.chemphys.2008.10.036

[S14] F. Weigend, Accurate Coulomb-fitting basis sets for H to Rn. Phys. Chem. Chem. Phys. **8**, 1057-1065 (2006). https://doi.org/10.1039/B515623H

[S15] H.M. Yu, D.P. Chen, Q.Y. Li, C.S. Yan, Z.H. Jiang et al., In Situ Construction of Anode–Molecule Interface via Lone-Pair Electrons in Trace Organic Molecules Additives to Achieve Stable Zinc Metal Anodes. Adv. Energy Mater. **13**, 2300550 (2023). https://doi.org/10.1002/aenm.202300550
